# Bone Marrow and Nonbone Marrow Toll Like Receptor 4 Regulate Acute Hepatic Injury Induced by Endotoxemia

**DOI:** 10.1371/journal.pone.0073041

**Published:** 2013-08-15

**Authors:** Edith Hochhauser, Orna Avlas, Reut Fallach, Larissa Bachmetov, Romy Zemel, Orit Pappo, Asher Shainberg, Ziv Ben Ari

**Affiliations:** 1 Felsenstein Medical Research Center, Rabin Medical Center, Petach Tikva, Israel; 2 Goldschmied Medical Diagnostic Research Center, the Mina and Everard Goodman Faculty of Life Sciences Bar-Ilan University, Ramat Gan, Israel; 3 Department of Histopathology, Sheba Medical Center, Tel Hashomer, Ramat Gan, Israel; 4 Liver Disease Center, Sheba Medical Center, Tel Hashomer, Ramat Gan, Israel; 5 Sackler School of Medicine, Tel Aviv University, Tel Aviv, Israel; Haassah Medical Center, Israel

## Abstract

**Background:**

Toll-like receptors (TLRs) are expressed in immune cells and hepatocytes. We examined whether hepatic Toll-like receptor 4 (TLR4) is involved in the acute hepatic injury caused by the administration of lipopolysaccharide (LPS) (septic shock model).

**Methods:**

Wild type (WT), TLR4-deficient and chimera mice underwent myeloablative bone marrow transplantation to dissociate between TLR4 expression in the liver or in the immune-hematopoietic system. Mice were injected with LPS and sacrificed 4 hours later.

**Results:**

Compared to TLR4 deficient mice, WT mice challenged with LPS displayed increased serum liver enzymes and hepatic cellular inflammatory infiltrate together with increased serum and hepatic levels of interleukin 1β (IL-1β), tumor necrosis factor α (TNFα) ,Up-regulation of hepatic mRNA encoding TLR4, IκB and c-jun expressions. TLR4 mutant mice transplanted with WT bone marrow were more protected than WT chimeric mice bearing TLR4 mutant hemopoietic cells from LPS, as seen by IL-1β and TNFα levels. We then used hepatocytes (Huh7) and macrophages from monocytic cell lines to detect TLR mRNA expression. Macrophages expressed a significantly higher level of TLR4 mRNA and TLR2 (more than 3000- and 8000-fold respectively) compared with the hepatocyte cell line. LPS administration induced TLR4 activation in a hepatocyte cell line in a dose dependent manner while TLR2 mRNA hardly changed.

**Conclusions:**

These results suggest that TLR4 activation of hepatocytes participate in the immediate response to LPS induced hepatic injury. However, in this response, the contribution of TLR4 on bone marrow derived cells is more significant than those of the hepatocytes. The absence of the TLR4 gene plays a pivotal role in reducing hepatic LPS induced injury.

## Introduction

The liver is considered important in the defense against bacterial infection. Endotoxin-induced acute liver failure is associated with high mortality [1]. Bacterial lipopolysaccharide (LPS), a major constituent of the outer membrane of gram-negative bacteria, is a powerful activator of the innate immune response and a major cause of septic shock. The liver plays an important role in LPS detoxification [2]. The gram-negative flora of the intestine is a substantial reservoir of endogenous LPS. LPS-induced activation of monocytes/macrophages leads to the secretion of a number of proinflammatory cytokines to eliminate bacterial infection [3]. Excessive cytokine production may result in fatal septic shock [4].

Recognition of many microbial toxins occurs through the activation of Toll-like receptors (TLR) that causes induction of the innate immune response [5,6]. Toll-like receptor 4 (TLR4) is specific for LPS from gram negative bacteria [5,7]. The signaling of TLR4 in response to LPS induces production of reactive oxygen species and expression of proinflammatory cytokines such as tumor necrosis factor-α (TNF- α), which is mediated by the activation of nuclear factor kB (NF-κB) [8]. Furthermore, TLR4-deficient and TLR4 mutant (C3H/HeJ) mice showed hypo-responsiveness to LPS, demonstrating that TLR4 is a critical receptor for LPS signaling [9,10]. Recent studies have provided evidence of TLR signaling in hepatic non-immune cell populations [5,11]. Primary cultured hepatocytes express mRNA for all Toll-like receptors (TLRs) and respond to TLR2 and TLR4 ligands, although hepatocytes express very low levels of TLR4, with weak responses in vivo [5,12,13]. Therefore, the contribution of hepatocyte TLR4 to hepatic inflammation following sensitization is unclear. Activation of Kupffer cells with the production of reactive oxygen species, up-regulation of proinflammatory cytokines and neutrophil accumulation, have all been identified as contributing events to the inflammation associated damage [8]. NF-κB and c-Jun N terminal kinase (cJUN) participate in the necrotic and apoptotic damage following liver ischemia reperfusion [14]. How initial cellular injury contributes to the propagation of the inflammatory response and tissue damage is poorly understood.

Cardiac function of TLR4 deficient (TLR4KO) and chimeric mice expressing TLR4 in the immune-hematopoietic system, but not in the heart, revealed resistance to LPS and reduced depression following MI, suggesting that TLR4 expressed by cardiomyocytes plays a role in this phenomenon [15].

We aimed to investigate the role of TLR4 in the regulation of the hepatic inflammatory reaction during endotoxemia. We examined the contribution of TLR4 in the hepatic inflammatory model of septic shock. We used a direct approach by LPS administration in wild type (WT) vs TLR4KO mice and the generation of chimeras by myeloablative bone marrow (BM) transplantation to attain TLR4 in the BM or liver. We showed that in TLR4 chimeric mice the whole liver recognizes LPS damage and activates TLR4 signaling required for LPS induced injury.

## Materials and Methods

### Ethics statement

All experiments were approved and performed according to the guidelines of the Tel Aviv University (Israel) Animal Care and Use Committee (IACUC no M-11-056). TLR4 deficient mice (C57BL/10ScN) were a generous gift from Dr. Stephan Jung, The Weizmann Institute, Rehovot, Israel.

### General experimental protocols

12-15 week-old male mice were divided into 2 groups (n = 24 each): TLR4KO (C57BL/10ScN) and WT controls (C57BL). Each group was divided into four subgroups (n=6 each): LPS treated and LPS non treated subgroups sacrificed at 4 and 24 hours following treatment.

Chimeras were generated by myeloablative BM transplantation to attain TLR4 only in the BM or only in the non hematopoetic systems (n=3) each group. Chimeric mice: WT/TLR4KO, TLR4KO/WT and WT/WT (donor/recipient) were LPS injected and sacrificed 4 hours following treatment.

### Septic-shock model LPS injection

Mice were injected i.p. with 10 mg/kg LPS inducing septic shock [15]. The LPS is a phenol extract of serotype 011:B4 L2630 (Sigma Chemical, San Diego, CA, USA). Control mice were injected i.p. with saline.

### Generation of chimeric mice

Chimerism was induced by myeloablative BM transplantation to dissociate between TLR4 expression in the immune-hematopoietic system and the liver. Mice (n=3 in each group), were grafted with 30x10^6^ whole BM cells (BMC) nucleated in 0.2 ml PBS into the lateral tail vein [16]. TLR4KO mice (H2K^b^, CD45.2) were grafted with BMC from WT syngeneic B6 donors (H2K^b^, CD45.1) (WT/TLR4KO) to express TLR4 in the immuno-hematopoietic system. To eliminate TLR4 expression in the immuno-hematopoietic system, WT B6 mice (H2K^b^, CD45.1) were grafted with BMC from syngeneic TLR4 KO donors (H2K^b^, CD45.2) (TLR4KO/WT). Control chimeras underwent transplantation and consisted of CD45.1 recipients and CD45.2 donors (WT/WT). Chimerism was determined at 8-10 weeks after BM transplantation. The fractional expression of donor and host peripheral blood lymphocytes used mAbs against minor antigens CD45.1 (cloneA20, eBioscience, Santiago, CA, USA) and CD45.2 (clone104 eBioscience, Santiago, CA, USA) with a Vantage SE-II flow cytometer (Becton Dickinson). Lymphocyte TLR4 expression was tested using Alexa Fluor® 488 anti-mouse Toll-like receptor 4 (CD284, TLR4) or with its isotype-matched irrelevant Ab (mouse IGg1 isotype control).

### Liver enzyme levels and cytokines

Aspartate transaminase (AST) and lactate dehydrogenase (LDH) were determined in the serum 4 hours post LPS-administration using commercial Olympus OSR6126 kit (Center Valley, PA, USA).

Serum and hepatic cytokines were assessed using ELISA semi-kits for mouse TNFα and mouse IL-1β (ELISA-Max, BioLegend, CA, USA) [15].

### Quantitative RT-PCR

Total RNA was purified from the livers by using TRIzol (Ambion, Austin, TX, USA) as per manufacturer’s instructions. The quantity of total RNA was determined by OD260 measurements. cDNA was synthesized from total RNA using the TaqMan High Capacity cDNA Reverse Transcription Kit (Applied Biosystems, Foster City, CA, USA) according to the manufacturer’s protocol.

Quantitative real-time PCR analysis for TLR4 was performed using the ABI 7000 Sequence Detection system (Applied Biosystems, Foster City, CA, USA). The primers and TaqMan FAM probes for TLR4 (Mm00445273_m1) and TATA-box (Mm00446973_m1) were ordered from Applied Biosystems. All samples were normalized to an endogenous gene, mouse TATA-box**.**

### Pathological evaluation

Specimens from livers in all groups were fixed in formalin, embedded in paraffin and stained with hematoxylin-eosin (H&E). Immunohistochemistry stain for rabbit anti-mouse TLR4 (1:100, Abcam, Cambridge, UK) was used. The anti-rabbit poly HRP detection kit (Chemicon, CA, USA) was used for TLR4 staining. Histological analysis was performed on livers from 5 mice for each group (10xHPF each), using ImagePro PLUS software (Media Cybernetics, USA). Pathological findings were assessed blind to group allocations [17].

The presence of myeloperoxidase (MPO) was used as an index of liver neutrophil accumulation [17].

### Western blot analysis of liver tissue

Liver tissue samples (20 mg) were homogenized and centrifuged at 8000 RPM for 10 min at 4^o^C in lysis buffer and quantified for protein levels using a Bradford reagent (Sigma, St. Louis, MO, USA). Liver extracts (30 µg protein/lane) were electrophoresed and subjected to SDS-PAGE under reducing conditions using 10% polyacrylamide gels. Proteins were transferred to nitrocellulose membranes and probed with the appropriate antibodies [10]. Mouse monoclonal antibody directed against the phosphorylated c-Jun NH2-terminal kinase [c-Jun(cc45) JNK] diluted 1:1000, rabbit anti-IκBα polyclonal antibody (the inhibitory protein of NF-κB) diluted 1:1000, and β actin diluted 1:10000, (loading control) polyclonal antibody were purchased from Santa-Cruz Biotechnology, Inc. (Santa Cruz, CA, USA).

### Hepatocyte cell culture preparation

Cell Culture Studies: Cells of human hepatocellular carcinoma cell line (Huh7) were maintained in complete growth medium (Dulbecco’s modified Eagle’s medium) supplemented with 10% fetal bovine serum, 2% penicillin/ streptomycin. Huh7 cells were stimulated with LPS (0.0625-0.5µ g/ml) for 4 hours, and then subjected to total RNA isolation. These concentrations have also been applied by others using a monocyte cell line (THP) and primary mouse hepatocytes using LPS 0.1-0.5µ g/ml [11,12]. These tests were conducted to show changes in TLR4 and TLR2 mRNA in purified hepatocytes after LPS challenge. Assay-on-Demand Gene Expression Products (Applied Biosystems) were used for the measurement of TLR2 (Hs00152932_m1), TLR4 (Hs00152939_m1), and GAPDH (Hs99999905_m1). The results are shown as the fold-change of the relative quantity of TLR mRNA normalized to GAPDH mRNA endogenous gene values. The control (non-treated) cells were assigned a value of 1. Results were normalized to control levels [18,19].

### Statistical analysis

Results are expressed as means±standard deviation. Differences between groups were assessed by the analysis of variance (ANOVA) with Bonferroni adjustment or by 2-tailed Student’s t test. The Kaplan-Meier method was used for survival. Differences were analyzed by the log-rank sum test. Statistical significance was defined as p < 0.05.

## Results

### Clinical manifestations and survival of sepsis

**Figure 1:**
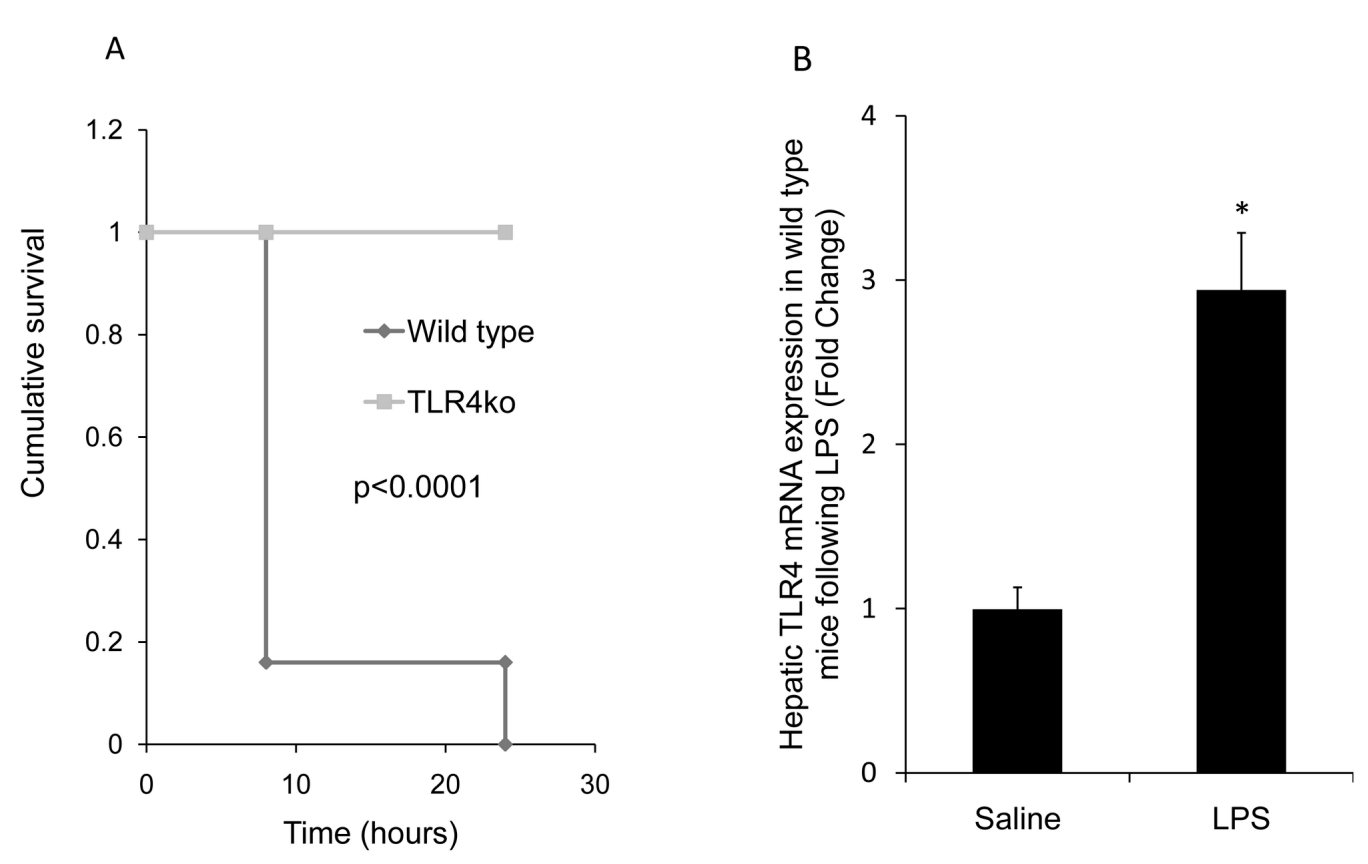
A: Kaplan-Meier survival curve. While 5/6 mice (83%) were treated with LPS died in less than 8 hours and the sixth died within the first 24 hours, all TLR4KO mice (6/6) survived following LPS (Kaplan-Meier, p < 0.001). **B: Real-time PCR analysis of hepatic TLR4 mRNA expression:** The results are shown as the fold-change of the relative quantity of TLR4 mRNA normalized to TATA-box mRNA values. TLR4 mRNA expression significantly increased 3-fold compared with saline administration in WT mice, 4 hours following LPS administration (*p ≤ 0.005); results are expressed as mean ±SE, n=4 in each group.

LPS challenged WT mice developed diarrhea, eye exudates and lethargy within 4 hours post LPS treatment, with 100% mortality within 24 hours. These symptoms were not observed in TLR4KO mice (Figure 1A, p < 0.0001, Kaplan-Meier).

## Hepatic TLR4 Gene and Protein Expression

To study the regulation of TLR4 expression in mouse liver, the animals were injected i.p. a high dose of LPS (10 mg/kg). Hepatic TLR4 gene expression increased almost 3 times in WT following LPS administration compared with saline administration at 4 h after the injection(p< 0.05) (Figure 1B). In TLR4KO mice no expression of the TLR4 gene was detected (data not shown).

### Liver enzymes

**Figure 2:**
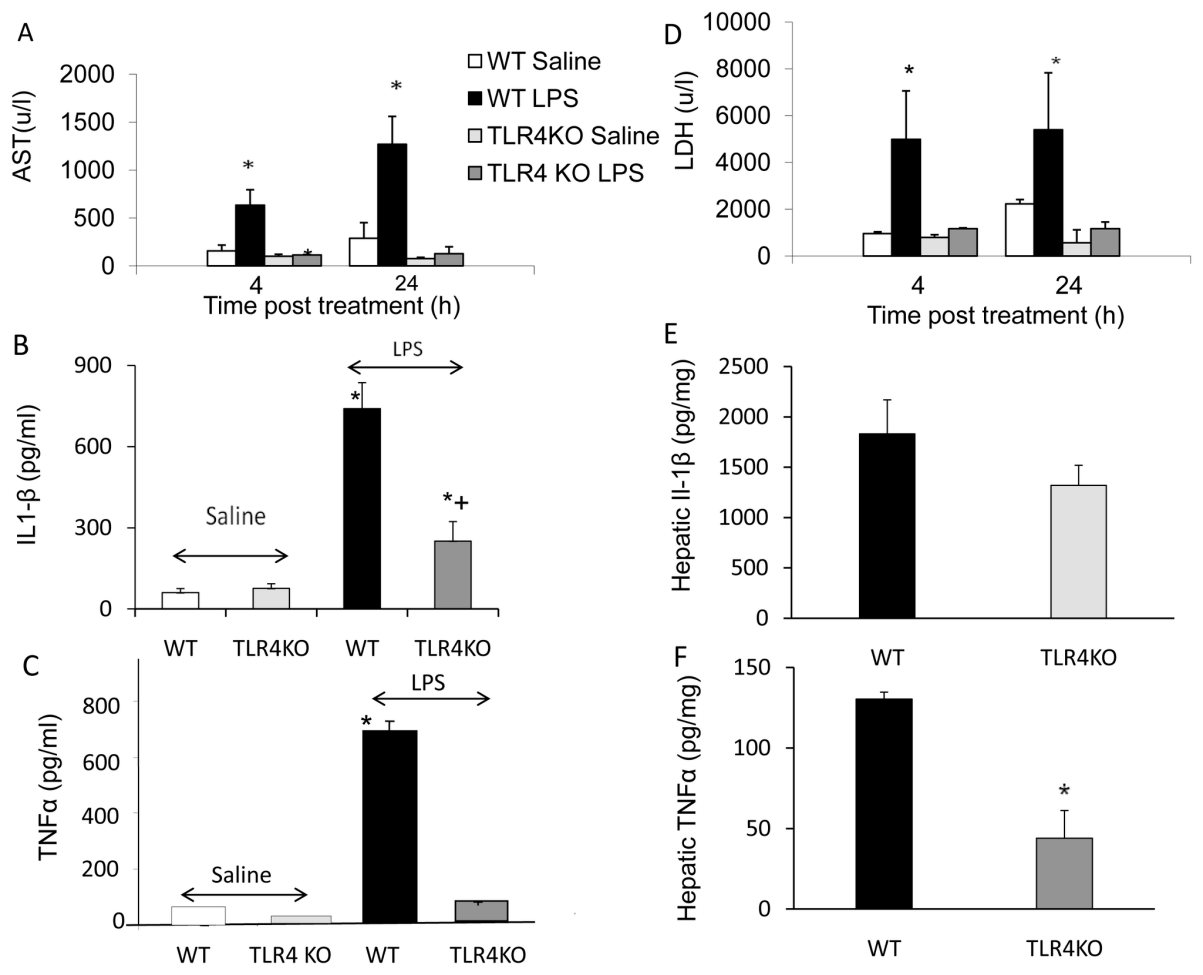
Serum biochemical analysis. Figs. A, D.: The serum LDH and AST level increased significantly in LPS-administered WT mice compared with saline administered mice. In LPS administered TLR4KO, the increase in serum enzyme levels was much lower compared with saline administered and compared with LPS administered WT mice (4 and 24 hours). Values are mean±SEM with n=6 mice per group *p<0.01 compared with other groups. Figs. B, E: The serum and hepatic Il-1β level increased significantly in WT mice following LPS compared with saline treated mice (p< 0.01). TLR4KO mice hepatic IL-1β level was significantly lower compared with WT LPS treated mice (p< 0.05). Values are mean±SEM (n=6 mice per group). Figs. C, F: The serum and hepatic TNFα level increased significantly in LPS WT-treated mice (p< 0.01). In TLR4KO mice the hepatic TNFα level was significantly lower compared with WT LPS treated mice (p< 0.005) and serum TNFα rose by a very small amount. Values are mean±SEM (n=6 mice per group).

Serum levels of AST, LDH increased significantly 4 and 24 hours post LPS administration in WT compared with TLR4KO mice (p< 0.01) (Figure 2A,D).

### Serum TNFα and IL-1β level after LPS challenge

The serum and hepatic IL-1β level increased significantly in WT compared with saline treated mice (p< 0.01). Although an elevation was seen in Il-1β level in TLR4KO mice, the hepatic IL-1β level was significantly lower compared with WT LPS treated mice (p< 0.05) (Figure 2B). While the serum and hepatic TNF-α level increased significantly in LPS WT treated mice (p< 0.01), in TLR4KO mice, levels were 3 times lower compared with WT LPS treated mice (p< 0.005). Serum TNFα was hardly elevated (Figure 2C).

### Histological studies

**Figure 3:**
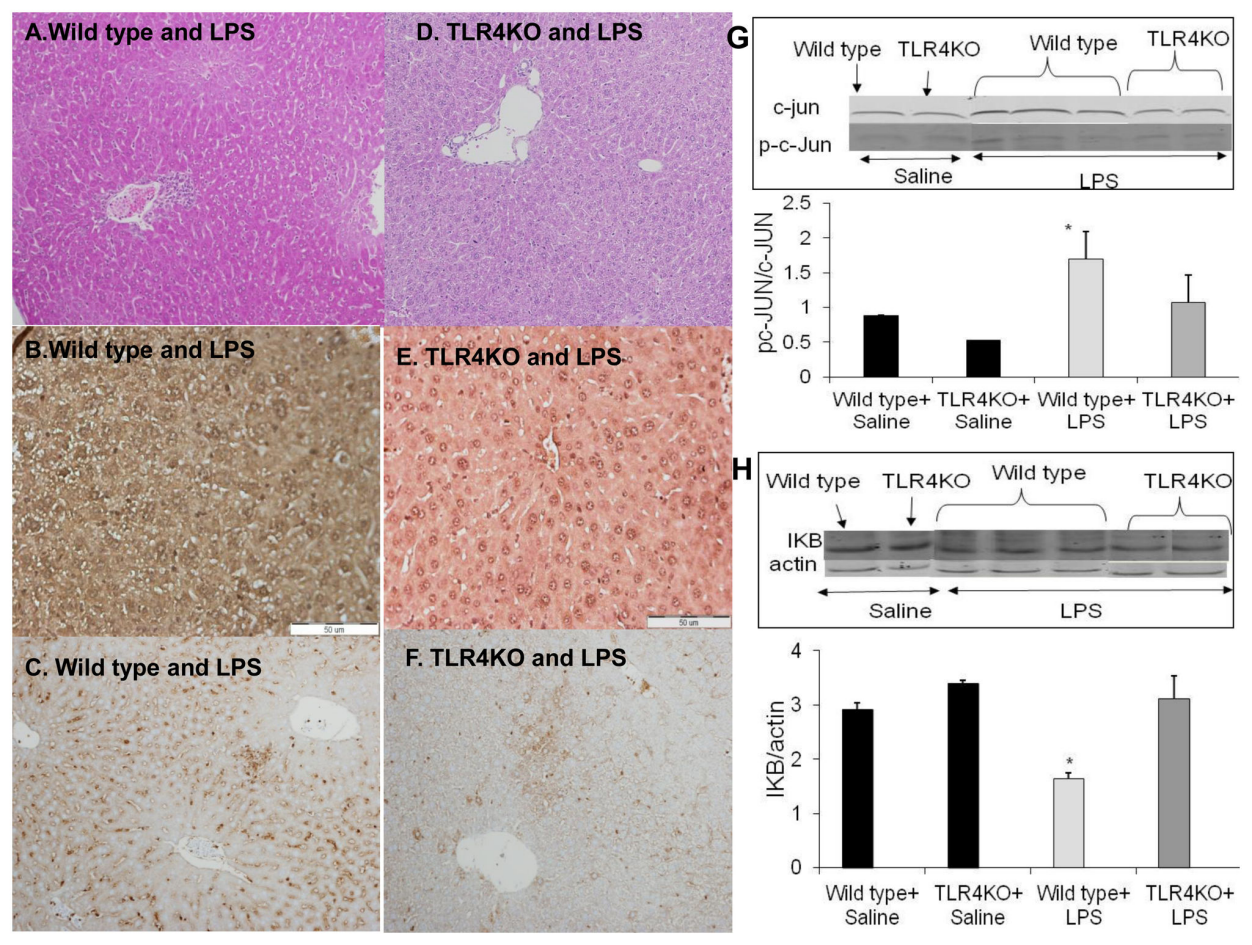
A, D: Histological staining using H&E. The inflammatory hepatic damage following LPS-administration in WT mice (A) compared to TLR4KO mice (D) was notably higher. **B, E: TLR4 protein immune staining in LPS treated mice:** TLR4 protein expression was upregulated at 4 hours (B) in WT mice compared to (E) no TLR4 immuno staining in TLR4KO. **C, F: MPO activity:** An enzyme specific for neutrophils in WT and in TLR4KO livers was examined. A significant increase in hepatic MPO activity was observed in LPS administered WT mice. In LPS administered TLR4KO mice, a slight increase in hepatic MPO activity was observed compared to WT liver. **G,H: Protein expression of phosphorylated c-JUN and IkB:** The hepatic expression of phosphorylated c-JUN was significantly higher in LPS administered WT mice compared with saline administered mice using Western blot, p<0.05. LPS administration increased c-Jun activity in WT mice compared to LPS treated TLR4KO mice, p<0.05 (G). The activation of the NFkB signaling pathway was higher in LPS administered WT compared to TLR4KO mice (H). IkB hepatic expression was significantly lower compared with saline administered mice, (p<0.05). In LPS administered TLR4KO mice there were no significant differences in the IkB hepatic expression between the LPS administered and the non LPS administered mice. Values are mean±SEM (n=6 mice per group).

Using H&E staining there was diffuse congestion and focal necrosis associated with neutrophil infiltration in the liver of WT mice LPS treated, after 4h, while in the TLR4KO mice only mild neutrophil infiltration was seen (Fig. 3A, D). Using Immunohistochemistry for TLR4 in WT mice the expression of TLR4 was upregulated at 4 hours after LPS administration as compared to TLR4KO liver tissue (Figure 3B, E). Determination of myeloperoxidase (MPO) activity a marker of neutrophil infiltration indicated a significant increase of MPO activity in LPS-administered WT mice compared to TLR4 KO mice (Figure 3C, F).

### The effect of LPS on the hepatic expression of phosphorylated c-jun and NFκB

The hepatic expression of phosphorylated c-JUN was higher in LPS administered WT mice than in saline-administered mice and exhibited more c-Jun activity (30%) compared to LPS treated TLR4KO mice (Figure 3G). IκB hepatic expression was 2-fold lower in WT compared with saline-administered mice (Figure 3H). In TLR4KO there was no significant difference in IκB hepatic expression between LPS administered and non LPS-administered mice.

### TLR4 in both BM derived cells and in endogenous liver cells contributed to LPS-induced liver injury

**Figure 4:**
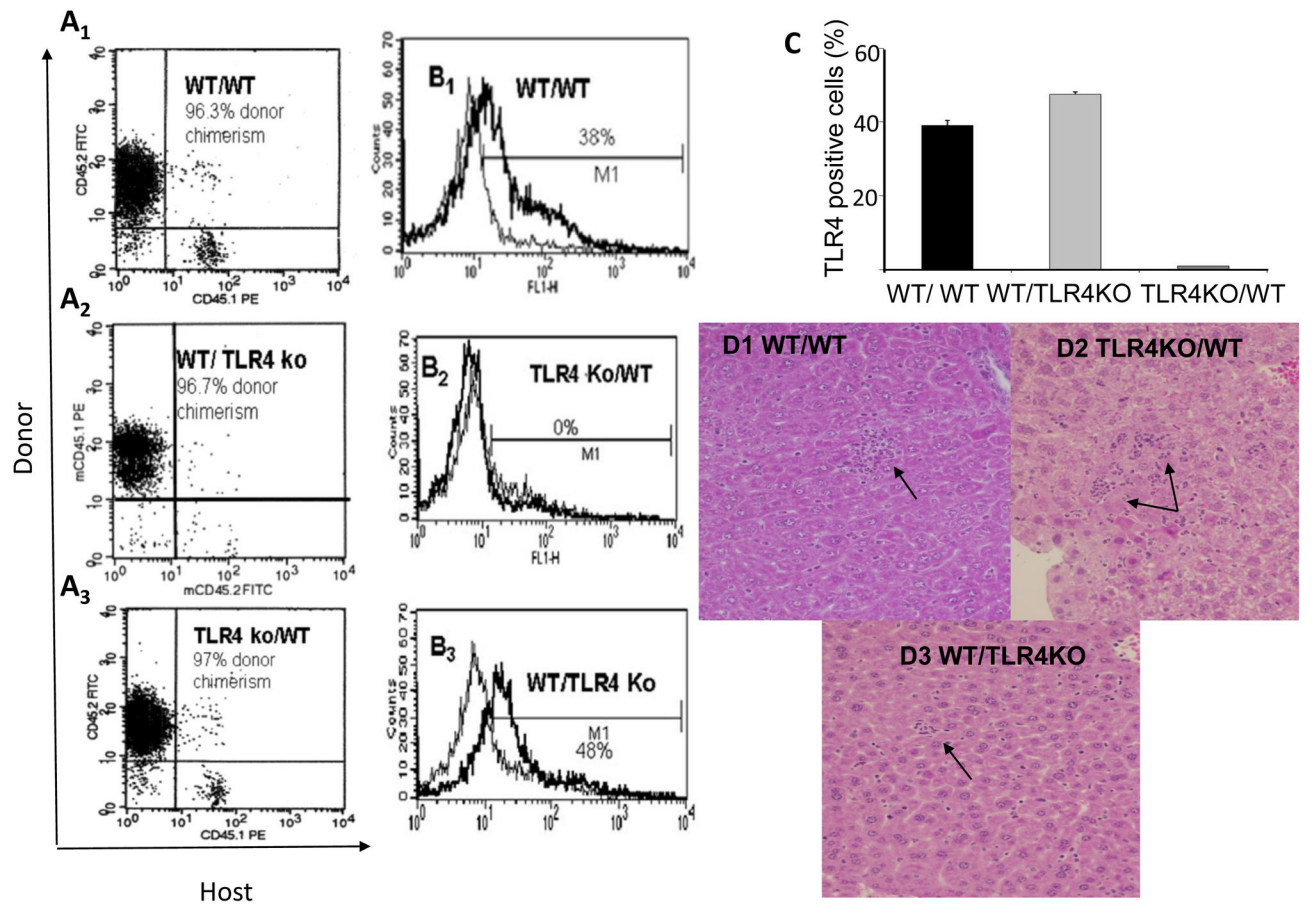
LPS challenge to hepatocytes and resident macrophages. To investigate the role played by hepatocytes and resident macrophages following LPS challenge, we used chimeric mice that expressed TLR4 either in the liver or in peripheral blood mononuclear cells. Chimerism was confirmed 8-10 weeks following transplantation when at least 95% of the transplanted bone marrow was present. Representative samples of the 3 transplanted groups: WT/WT, TLR4KO/WT, WT/TLR4KO mice are presented in Figure A1 –A3. Following LPS challenge, TLR4 was highly expressed in the leukocyte–immune bone marrow derived cells in both chimeric mice that were transplanted with WT BM, as analyzed by FACS and presented in Figures B1 and B3. However, TLR4 expression was not induced in the leukocytes following LPS in chimeric mice that were transplanted with TLR4KO BM, since the hematopoietic system lacks TLR4 as presented in B2. TLR4 expression in the different groups are stated as mean ±SEM (n=3 in each group) and presented in Figure C. Histological analysis of chimeric mice challenged with LPS is presented in Figure D1-D2. There was diffuse hepatic congestion and focal necrosis associated with diffused neutrophil infiltration in LPS administered WT/WT, TLR4KO/WT mice compared to WT/TLR4KO mice administered LPS in which few neutrophils were observed (D3). Values are mean±SEM (n=3 mice per group) *p>0.05 compared with other groups.

To investigate the role of TLR4 expressed in the acute phase (4 hours) on BM derived cells compared with endogenous liver cells, chimeric WT mice transplanted with TLR4KO BM and TLR4KO mice transplanted with WT BM were used. Figure 4A1–A3 are representative samples of the 3 transplanted groups: WT/WT, TLR4KO/WT, WT/TLR4KO mice. In the chimeras the donor congenic marker is dominant in at least 95% of the events. No death occurred at 4 hours following LPS treatment in the chimera mice.

Following LPS challenge, TLR4 was highly expressed in the leukocyte–immune BM derived cells in both chimeric mice (Figure 4B1 and B3). However, since the hematopoietic system lacks TLR4, its expression was not induced in leukocytes following LPS in chimeric mice that had been transplanted with TLR4KO BM, (Figure 4B2).

Histological analysis of mice challenged with LPS demonstrated that in WT/WT, TLR4KO/WT mice, diffuse hepatic congestion and focal necrosis associated with diffused neutrophils infiltration was noted (Figure 4D1–D2) compared with LPS administered WT/TLR4KO mice in which few neutrophils were observed (Figure 4D3).

**Figure 5:**
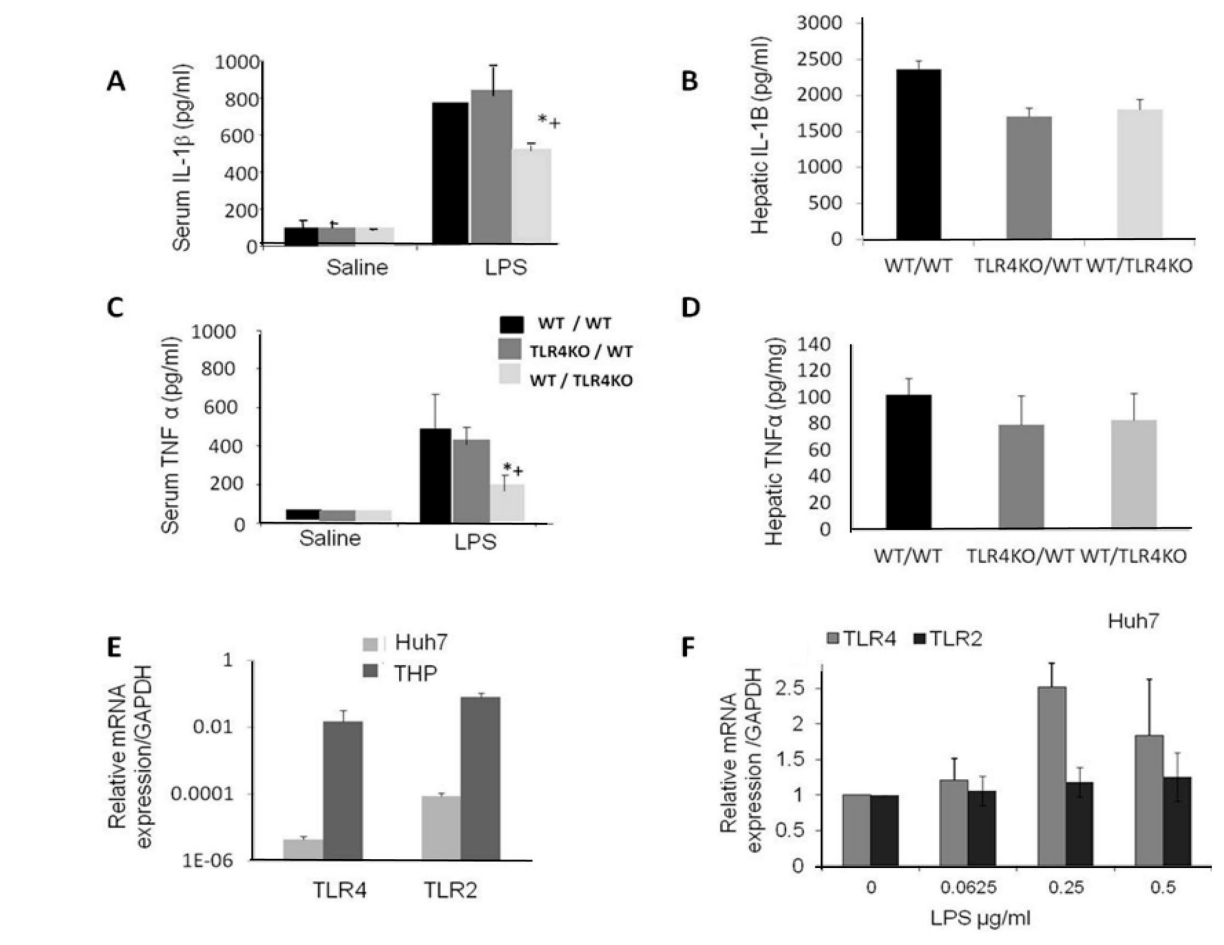
A,C: TNFα and IL-1β protein expression in chimeras in serum and liver. In all chimeras, both TNFα and IL-1β increased significantly at 4 hours post LPS challenge. The proinflammatory cytokine levels in chimeras expressing TLR4 in the liver were found to be significantly higher than the levels expressed in TLR4KO chimera livers ( * p<0.05 vs. WT/WT LPS treated ; + p<0.01 vs. + WT/WT saline-treated). Values are mean±SEM ( n=6 mice per group). **B,D: Hepatic TNFα and IL-1β expression:** No differences were observed between hepatic TNFα and IL-1β whether the livers expressed TLR4 or KO for TLR4. Values are mean±SEM (n=3 mice per group). **E: TLR2 and TLR4 mRNA expression level in a Huh7 cell line:** Macrophages expressed a significantly higher level of TLR4 and TLR2 mRNA compared with the hepatocyte cell line (p < 0.0001). **F: TLR2 and TLR4 mRNA expression following LPS treatment:** LPS induced TLR4 activation in a hepatocyte cell line in a dose dependent manner while there was no change in TLR2 mRNA expression. The results are shown as the fold-change of the relative quantity of TLR mRNA normalized to GAPDH mRNA values; the control cells were assigned a value of 1 and results are normalized to controls. Each point represents the mean ±SEM of a set of data determined by at least 5 experiments.

In all chimeras, both TNFα and IL-1β were elevated significantly at 4 hours post-LPS challenge. However, the proinflammatory cytokine levels in chimeras expressing TLR4 in the liver were found to be significantly higher than the levels expressed in TLR4KO chimera livers (Figure 5A, C, p<0.05). However, in the liver tissue of the chimeras no differences were observed between livers expressing TLR4 or KO for TLR4 (Figure 5B, D). We analyzed TLR4 gene expression in a hepatocyte (Huh7) and a macrophage cell line (THP). TLR2 was included as another TLR mRNA and served as a control. Macrophages expressed a significantly higher level of TLR4 mRNA and TLR2 (more than 3000- and 8000-fold respectively) compared with the hepatocyte cell line. Administration of LPS to the hepatocyte cell line induced TLR4 activation in a dose dependent manner with no change in TLR2 mRNA expression. These results supports the notion that hepatocyte TLR4 also participates in the response to LPS. However, the contribution of hepatocytes might be less dominant than macrophages (Figure 5F).

## Discussion

**Figure 6:**
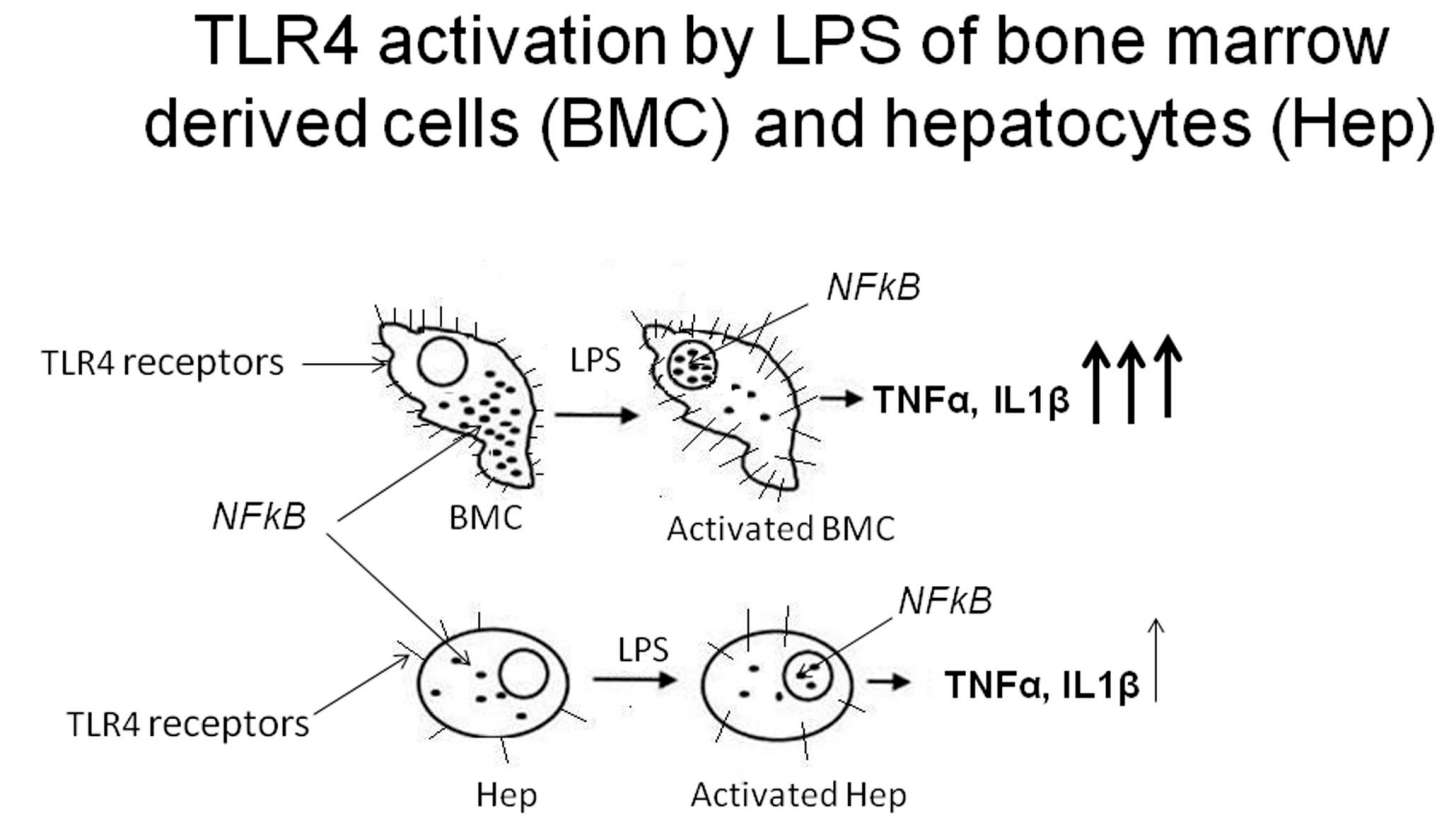
BMC and hepatocytes following TLR4 activation by LPS. A schematic representation of the relationship between bone marrow derived cells (BMC) and hepatocytes (Hep) following TLR4 activation by LPS. While in the bone marrow both TLR4 and NFκB are highly expressed in the hepatocytes, the expression level is markedly low. The marks on the cell membrane are TLR4 receptors, the structures (dots) describe NFκB molecules in the cytosole. When exposed to LPS, TLR4 receptors are activated to induce NFκB translocation to the nucleus resulting in the secretion of cytokines such as TNFα and IL1β. The contribution of TLR4 BMC is more significant than the hepatocytes in this response, as seen by the amount secretion of different cytokines.

This study evaluates the contribution of hepatic TLR4 under conditions of septic shock when liver transplantation is often mandatory. Simulation of shock by LPS administration induced the decreased survival of WT mice. Our results suggest that although TLR4 activation of BM derived cells is a central player in the immediate response to LPS induced hepatic injury, both TLR4 in BM and in the hepatocytes were activated in the LPS response. A schematic representation of the relationship between bone marrow derived cells and hepatocytes following TLR4 activation by LPS is presented in Figure 6.

Hepatic cytokine response to LPS was remarkably rapid (4 hours) in the liver and liver macrophages secrete proinflammatory cytokines, including IL1-β and TNFα known to be the terminal mediator of hepatic organ failure [20,21]. These results reveal the important role for the liver as a cytokine producing organ during the early phase of endotoxemia or I/R injury, suggesting that the primary activation of the cytokine cascade occurs in the liver [22] and hepatic injury is less in TLR4KO mice following LPS administration or I/R injury.

We and others also found that the inflammatory response and the liver injury in TLR4KO mice was significantly attenuated through decreased hepatic c-Jun phosphorylation and NFκB activation and thus decreased cytokine production in GalN/LPS-induced FHF [10,23]. NFκB activation was clear in Kupffer cells and in hepatocytes during LPS-challenge, suggesting these cells are the main source of *in vivo* cytokine production [20]. Both NFκB and activation of c-Jun phosphorylation contribute to the inflammatory response of the host defense against pathogens.

We used hepatocyte and macrophage cell lines since a cell line is a more homogenous population than isolated primary cells. Based on our unpublished results, primary cell culture contains residual CD45 positive cells [24]. Measuring TLR4 expression in hepatocyte and macrophage cell lines revealed that macrophages expressed a significantly higher level of TLR4 mRNA compared to the hepatocyte cell line. mRNA for TLR4 was also elevated in ventricular myocytes as well as in the liver following LPS challenge both *in vitro* and *in vivo* [14,25,26]. Immunohistochemistry for TLR4 protein expression demonstrated a parallel increase of protein expression following LPS administration. These results are in contrast to the previous study of Ojaniemi (20) in which a significant cytokine response following i.p. LPS (serotype 055:B5) injection but without parallel TLR4 protein expression, were found [20]. Perhaps the use of a different LPS serotype or a different protein detection technique is responsible for these diverse results. However, robust interleukins production together with TLR4 activation (mRNA) was noted in the lungs 2 hours post LPS administration [20]. The liver, similar to the heart or lungs, responds to LPS with TLR4 upregulation. Our chimeric mice that were TLR4 positive in the organs, secreted a large amount of cytokines through different organs of the body to the serum, as a result of LPS activation.

Isolated Kupffer cells and hepatic endothelial cells exposed to LPS are able to produce inflammatory cytokines [27] and to modulate the host reactions to pathogens. Our *in vitro* and *in vivo* results indicate that TLR4 in both hepatocytes and macrophages participate in this response to LPS. However, the contribution of hepatocytes to this response might be less dominant.

In conclusion, the relative contribution of TLR4 activation on bone marrow and nonbone marrow cells to the inflammation process, together with the better survival of TLR4KO mice after LPS suggest that TLR4 activation plays a pivotal role in hepatic LPS induced injury. Inhibition of TLR4 signaling pathway in the various liver cells may be beneficial in overcoming the acute phase. Anti-TLR4 antibodies were shown to prevent death caused by septic shock, even when treatment was given 4 hours after the LPS challenge [28]. We demonstrated that in the acute phase, hepatic injury in response to LPS is directly mediated by the immune cells together with the hepatocytes. TLR4 might be a therapeutic target to prevent and block suppression of hepatic function under conditions of septic shock.
